# Evaluation of the coverage and effective use rate of long-lasting insecticidal nets after nation-wide scale up of their distribution in Benin

**DOI:** 10.1186/1756-3305-6-265

**Published:** 2013-09-16

**Authors:** Filémon T Tokponnon, Bruno Aholoukpe, Eric Y Denon, Virgile Gnanguenon, Alexis Bokossa, Raphael N’guessan, Mariam Oke, Dorothée Kinde Gazard, Martin C Akogbeto

**Affiliations:** 1 National Malaria Control Program, Cotonou, Benin; 2 Ministry of Health, Cotonou, Benin; 3 Faculte des Sciences et Techniques de l’Université d’Abomey-Calavi, Abomey-Calavi, Benin; 4 Centre de Recherche Entomologique de Cotonou (CREC), Cotonou, Benin; 5 London School of Hygiene and Tropical Medicine, Kepel Street, London, UK

**Keywords:** Long-lasting insecticide-treated nets, Universal coverage, Malaria, Benin

## Abstract

**Background:**

In Benin, around four million Long-Lasting Insecticide-treated Nets were freely distributed to household to prevent malaria in 2011. In contrast to a previous campaign that targeted only children under 5 years and pregnant women, this distribution campaign was conducted in order to achieve universal coverage. This study presents the results of LLIN coverage and utilization after the distribution campaign.

**Methods:**

The study was a cross-sectional household survey which utilized a stratified two-stage cluster sampling design. The strata represented the twelve departments covered by the national distribution campaign in 2011 and included a total of 4,800 households randomly selected in the country. A questionnaire adapted from the standard Malaria Indicator Survey (MIS) Household Questionnaire was used. Data were entered using QPS software and analyzed with R 2.14.1.

**Results:**

LLIN ownership was 86.4% (74 – 94). On average, each household received 3 LLINs (2–4). The proportion of households that met the ratio one net for two persons was 77%.

The proportions of individuals sleeping under LLINs were high (84.8%). LLIN use among urban residents was 10% lower than in effective users from rural areas (P = 0.00224).

**Conclusions:**

The universal distribution campaign conducted in Benin has increased LLIN ownership and use in the community. But additional efforts are need to improve and maintain LLIN coverage.

## Background

The scaling- up of LLINs interventions to achieve high coverage of most or ultimately all at-risk populations has become the national malaria control standard in the Africa region with substantial support from the Global Fund and the Roll Back Malaria (RBM) Partnership [1]. Insecticide-treated nets (ITNs) remain effective tools for malaria prevention and can significantly reduce severe disease and mortality due to malaria, especially among the most vulnerable populations [2]. In recent decades, resources to fight malaria have increased and many countries across sub-Saharan Africa are rapidly expanding LLIN ownership coverage through several strategies including, social marketing [3,4] and free distribution to target groups (through antenatal care or immunization campaigns) [5-9]. Actually, promotion of LLIN use has shifted in emphasis from a focus on target groups to a broader objective of universal coverage. To achieve universal coverage, the RBM Partnership aim to distribute one LLIN for every two people by 2015 [1]. The main goal of this mass distribution is to protect all people living in endemic areas [10].

In 2007, the first campaign of mass distribution of LLINs took place in Benin. The targets were children under 5 years and pregnant women. Since 2008, Benin has adopted a strategy of routine distribution of LLINs to children <1 year old through measles vaccination sessions and pregnant women through antenatal clinics. In order to achieve universal coverage, the National Malaria Control Programme (NMCP) distributed 4 674799 new LLINs mainly Olyset Nets® from Summitomo, PermaNet 2.0® from Vestergaard-Frandsen, and Interceptor® from BASF, all free of charge and accessed through a campaign in 2011. The new National Strategic Plan to control malaria will repeat this intervention every three years to maintain universal coverage for sustained impact against malaria. Several partners are involved in the implementation of this intervention, including the Global Fund to Fight AIDS, Tuberculosis and Malaria (GFATM), the World Bank, the USA President’s Malaria Initiative (PMI) and Projet d’Appui au Développement du Système Sanitaire (PADS)/WHO. To supplement LLINs, yearly rounds of Indoor Residual Spraying (IRS) based on bendiocarb (FICAM® WP, BAYER,) were done on a regional basis.

The urgent need for data to inform policy about the level of achievement of the universal coverage led to the decision to conduct a rapid assessment of the first campaign for universal access. In this we aimed to determine the proportions of households that received free distribution of LLINs during the campaign and estimate households’ coverage rate at the national and regional level after the campaign. We also evaluated the proportions of children under five and pregnant women using LLINs.

## Methods

### Study area

The study was conducted in Benin (West Africa) and involved 12 districts

Alibori, Borgou, Atacora and Donga from the North of the country

**Figure 1:**
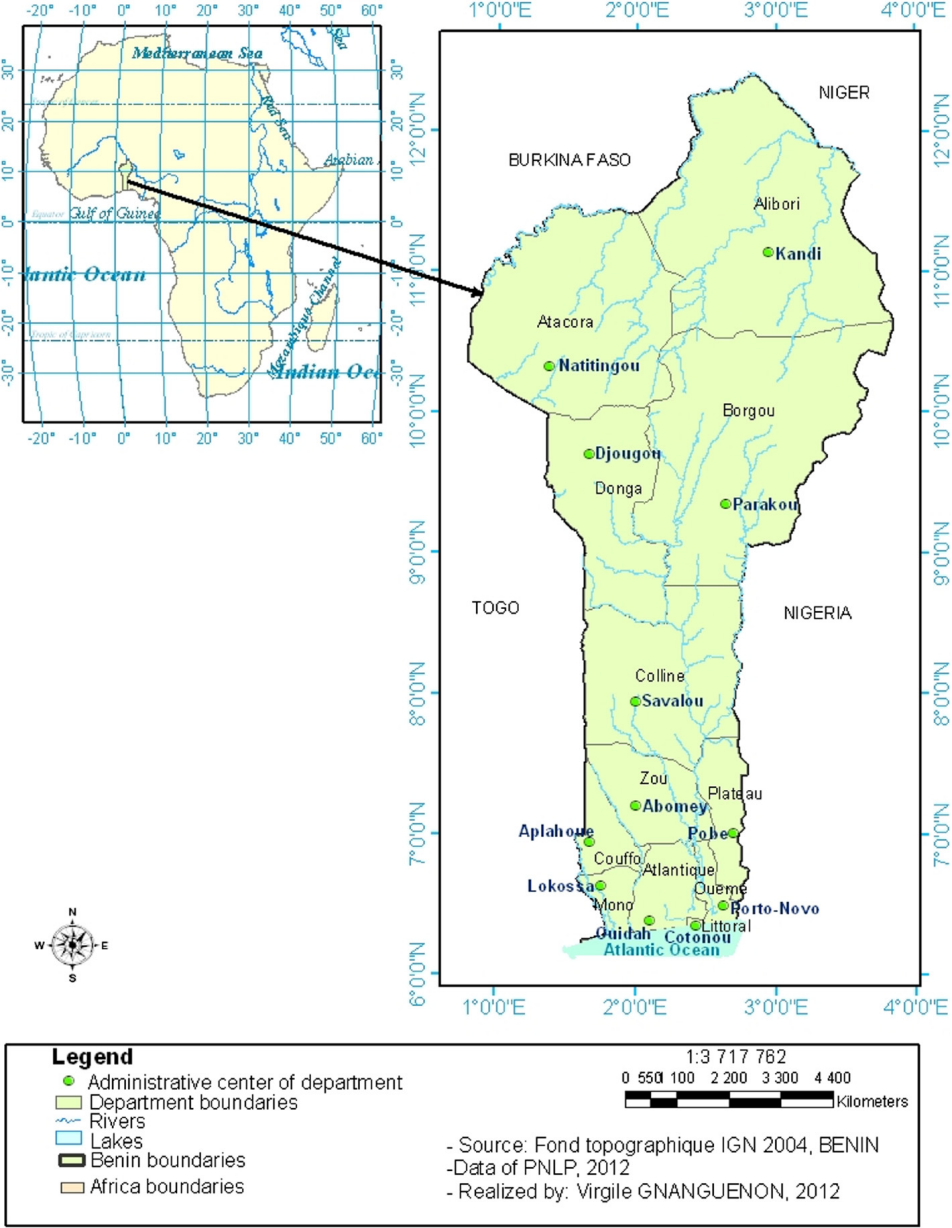
Study area.

Collines, Zou in the center;

Couffo, Plateau, Mono, Atlantique, Oueme and Littoral located in the southern part (Figure 1). The northern area is characterized by a Sudanian semi-arid bioclimatic zone with only one rainy season in the year (June to October) and a mean annual rainfall below 900 mm. It is characterized by a dry savanna.

The center part is characterized by an intermediate bioclimatic zone (tropical Sudano-Guinean climate) with humid savanna and an average rainfall reaching 1000 mm per year.

The southern area, near the Atlantic coast, is characterized by a Guinean-bioclimatic zone with two rainy seasons (April–July and September–November) and an average annual rainfall of >1500 mm with degraded tropical forest.

### Study population

The study was a cross-sectional household survey which utilized a stratified two-stage cluster sampling design. The strata represented the twelve departments covered by the national distribution campaign in 2011. Each district represented a survey domain. The first stage was the selection of the clusters. In each district, an alphabetical list of villages and grouping of populations sizes by village and city were established. A total of 40 clusters were selected per district. Within each cluster, 10 households were randomly selected, resulting in a total sample of 4,800 households in the country. To form clusters, population data was obtained from the third General Population and Housing Census conducted in Benin in 2002.

The second stage involved the selection of households. In the cluster, the first household to investigate was randomly selected from the household registration lists of the distribution campaigns. The first household found in this list was the gateway to the cluster. In urban areas with more houses, the selection of the next households were done after every 10 households count while in rural areas a reduced jump space of 5 households was left between two consecutive households selected.

### Recruitment and training of interviewers

Interviewers were recruited by district on advice and recommendation by regional health officers. Four interviewers and one supervisor were recruited per district. Interviewers were nurses, midwives, social health workers and anthropologists, while supervisors were senior consultants. In order to avoid information bias, interviewers were deployed in a department other than the one they originate from. Before the collection of information in selected clusters, a training session was organized by interviewing 60 households from 12 villages located in districts near Cotonou, the capital city of Benin, not included in the study. Adjustment was made after the training of the field team.

### Data collection

Data collection was conducted from November 28th to December 5th, 2011, corresponding to four months after the distribution campaign. A questionnaire adapted from the standard Malaria Indicator Survey (MIS) Household Questionnaire [11] was used. The questionnaire was divided into sections including household roster, household characteristics, the campaign of net distribution, nets received during the campaign, nets owned by the household and their use.

In each district, the data were collected by two teams. Each team was composed of two interviewers and a supervisor. In each cluster, interviewers were aided in their task by a guide identified locally in collaboration with the head of the village or the officer of the health center. Each team covered an average of 2–4 clusters per day depending on the location.

Each selected household was visited. The head of household or an adult person acting on behalf of the head was interviewed. In occasions where no appropriate respondent was found in a particular household, the visit of the next home was scheduled. The techniques used for the data collection are structured interviews and direct observation after obtaining the consent of the head of household. A quality control check validating the data collected was made by each supervisor. A summary report form on the quality of the data collected was developed. It aims to check for the compliance with the household recruitment methodology and the completeness of the survey forms.

### Data processing and analysis

Data entry was done using QPS software with double entry of all records. Both data sets were then compared and any discrepancy in records was verified using the original questionnaires.

After the first stage of cleaning, the data set was transferred to R 2.14.1 for analysis.

### Indicators

Net coverage: the proportion of households with at least one LLIN and the average number of LLIN per household.

Ratio one net for two person (Intra-household coverage): the proportion of household with at least one LLIN for two people.

Net usage: proportion of residents who slept under LLIN the previous night.

The association between LLIN ownership, usage and explanatory variables (E.g. Size of households, gender, pregnancy status, etc.) were assessed using a logistic regression.

### Ethical clearance

This paper used data from net free distribution campaign survey conducted on behalf of the National Malaria Control Programm in Benin. Because this was part of the programmatic activity, ethical clearance was exempted. Informed consent was obtained from each participant.

## Results

### Sample characteristics

A total of 4800 households participated in the survey (Table 1). Of these, 2991 representing 62% lived in rural areas and 1809 (38%) in urban areas. Among the heads of households interviewed, 2173 (45%) were female and 2627 (55%) were male. In the rural areas 41% of the heads of households were female and 59% were male whereas in the urban areas 53% of the heads were female and 47% were male. The average household size was 6 people (Table 1). Children under the age of five were recorded in 66% (3182) of the households and the average number of children under five per household was 1. Pregnant women were recorded in 14% (652) of the households (Table 1).

**Table 1:** Baseline characteristics of households

	**Number of households**	**Average size of households**	**Average households with at least one child < 5**	**Average children under 5 per households**	**% households with at least one pregnant woman**
**Atacora**	400	6 [5.89-6.72]	65 [63.86-66.14]	1 [0.95-1.15]	10 [09.03- 11.04]
**Donga**	400	7 [6.91-7.42]	72 [70.83-72.67]	1 [1.27-1.53]	13 [12.02-14.04]
**Alibori**	400	5 [5.09-6.36]	63 [62.06-64.44]	1 [0.94-1.13]	14 [13.02- 15.03]
**Borgou**	400	10 [8.95-12.68]	67 [66.19-68.31]	2 [1.62-2.10]	19 [18.02- 20.02]
**Collines**	400	6 [6.04-7.29]	74 [73.16-74.85]	1[1.11-1.31]	17 [16.03-18.00]
**Zou**	400	5 [5.17-6.97]	60 [58.0-61.30]	1 [0.82-1.00]	12 [11.04-13.02]
**Mono**	400	6 [5.46-6.60]	63 [61.54-63.96]	1 [0.90-1.09]	14 [13.03-15.02]
**Couffo**	400	9 [8.17-10.92]	72 [70.83-72.67]	2 [1.40-2.71]	18 [17.03-19.01]
**Ouémé**	400	5 [5.12-6.17]	64 [63.09-65.41]	1 [0.95-1.53]	13 [12.02-14.03]
**Plateau**	400	6 [5.63-7.32]	62 [60.25-62.75]	1 [0.92-1.13]	10 [09.04-11.03]
**Atlantique**	400	7[6.50-7.89]	72 [71.09-72.91]	1 [0.88-1.38]	15 [14.03-16.01]
**Littoral**	400	5 [5.06-6.11]	62 [60.77-63.24]	1 [0.82-1.00]	11 [10.01- 12.05]
**Mean average**	**400**	**6.42 [6.04-6.78]**	**66.33 [65.99-66.67]**	**1.16 [0.94-1.39]**	**13.83 [13.37-14.28]**

### ITNs coverage and ownership after the distribution campaign in July 2011

Of the 4800 households surveyed, 4672 (97.3%) were informed of the distribution campaign. LLINs distribution vouchers had been given to 4290 (89.4%) households and 4147 (96.7%) of them actually received LLINs from the campaign (Table 2). LLINs universal coverage targeted by the distribution campaign was not met in any department. The percentage of households that met the universal coverage rate is lower in Littoral (52.5%) and Oueme (73%) and higher in Collines and Borgou (86%)”. The proportion of individuals sleeping under LLINs was 84.8%, above the threshold of massive usage. The distribution campaign increased the LLIN coverage (proportion of households with at least one LLIN) and usage (Table 2). The universal coverage was not reached in all the households but the LLINs use by people was above the threshold (80%) leading to a collective protecting effect (mass protection effect).

**Table 2:** LLINs coverage and ownership per household

**Department**	**% of households informed about the distribution**	**% of households that received a coupon**	**% of household that received LLIN**	**Average LLIN received per household**	**Proportion of households that met the ratio one net for two persons**
**Atacora**	97.0 [94.83-98.28]	95.7 [93.30-97.33]	93.8 [90.94-95.73]	2.98	75.3 [71.02-79.47]
**Donga**	97.0 [94.83-98.28]	96.7 [94.52-98.09]	94.8 [92.11-96.54]	3.26	83.3 [79.60-86.90]
**Alibori**	99.0 [97.46-99.61]	91.3 [88.07-93.64]	89.3 [85.83-91.92]	2.36	75.5 [71.28-79.71]
**Borgou**	99.5 [98.20-99.86]	93.2 [90.36-95.32]	89.5 [86.11-92.14]	3.95	86.0 [82.59-89.40]
**Collines**	98.3 [96.43-99.15]	92.2 [89.21-94.49]	92.3 [89.21-94.49]	2.94	86.3 [82.87-89.62]
**Zou**	98.7 [97.46-99.61]	89.2 [85.83-91.92]	86.3 [82.53-89.28]	2.34	77.5 [73.40-81.59]
**Mono**	91.5 [97.46-99.61]	84.2 [80.36-87.49]	79.3 [75.01-82.94]	2.27	78.0 [73.94-82.05]
**Couffo**	96.0 [88.36-93.61]	89.0 [85.55-91.70]	88.5 [85.00-91.27]	3.36	79.3 [75.27-83.22]
**Oueme**	96.7 [94.52-98.09]	80.3 [76.07-83.86]	76.8 [72.37-80.62]	1.98	73.0 [68.64-77.35]
**Plateau**	98.0 [96.10-98.98]	87.3 [83.62-90.17]	85.5 [81.71-88.61]	2.70	79.8 [75.81-83.68]
**Atlantique**	99.2 [97.82-99.74]	91.5 [90.36-95.32]	87.0 [83.35-89.95]	2.78	80.8 [76.88-84.61]
**Littoral**	97.0 [94.83-98.28]	81.7 [77.67-85.23]	74.0 [69.49-78.06]	2.00	52.5 [47.60-57.39]
**Mean**	**97.3 [96.84-97.75]**	**89.4 [88.47-90.2]**	**86.4 [85.40-87.34]**	**2.74**	**77.3 [76.06-78.43]**

### Factors associated with LLINs ownership

In the urban areas, 1456 (80.49%) households out of a total 1809 households had at least one LLIN. In the rural areas, 2691 households representing 89.97% of the households had at least one LLIN (Table 3). The area of residence, i.e. urban residents had 54% lower opportunity to own an LLIN than those living in rural areas (OR = 0.46 [0.39-0.54]; p < 0.001) (Table 3).

**Table 3:** Logistic regression assessing factors associated with LLIN ownership

**Factors**	**Number of individuals**	**Number of individual using LLIN (%)**	**Odds ratios (95% CI)**	**P**
Total number of individuals	27794	23575(84.82)		
**Place of residence**				
Rural	9458	15640(85.30)	1	
Urban	18336	7935(83.90)	0.90 [00.84-00.96]	0.00224
**Size of the household**				
0-1 member	145	131(90.34)	1	
2-4 members	3915	3490(89.14)	0.97 [00.53-01.64]	0.91232
5-7 members	8876	7692(86.66)	0.89 [00.49-01.50]	0.68577
8 and more members	14858	12262(82.53)	0.80 [00.44-01.35]	0.43840
**Age**				
15-25 years	2523	2143(84.94)	1	
26-35 years	7207	6385(88.59)	1.50 [01.31-01.72]	< 0.001
36-45 years	7741	6721(86.82)	1.34 [01.17-01.53]	< 0.001
46 and mores years	10323	8326(80.65)	0.91 [00.80-01.03]	0.12776
**Ratio 1 net for 2 person met**				
No	13174	10333(78.43)	1	
Yes	14620	13242(90.57)	2.47 [02.30-02.65]	< 0.001
**Gender**				
Female	10161	8630(84.76)	1	
Male	17633	14945(84.93)	1.10 [01.03-01.19]	0.855

Crowding within households played a significant role in ownership of LLINs. The odds of securing an LLIN at home during the campaign increased steadily with family members found within households. Relative to households with a single occupant, it significantly increased to 1.52 [1.03-2.23] (P = 0.034) within households with 2–4 members, 1.78 [1.2-2.63] (P = 0.004) in those with 5–7 members and to 2.66 [1.75-4.06] (P < 0.001) in households sharing 8 members and above.

There was no association between ownership of LLINs and the status of being pregnant at a district (OR = 1.13 [0.87-1.48]; P = 0.728). By contrast, both the head of household gender status of being a male and the fact of having a child <5 yrs old within households significantly increased the chance of owning an LLIN (OR = 1.53 [1.27-1.83]; P < 0.001 for gender) and (OR = 1.39 [1.14-1.7]; P = 0.001 for age) (Table 3).

### LLINs actual use rate and associated factors

The proportions of individuals sleeping under LLINs the night before they were interviewed was high (84.8%). The odds of effective use of LLINs among urban residents was 10% lower than in effective users from rural areas (OR = 0.90 [00.84-00.96]; P = 0.00224).

The actual usage rates of LLINs among family members were similar across districts, regardless of whether members were crowded or not within households (0R = 0.80-0.97; p = 0.43-0.91) (Table 4). Middle age class (25–46 yrs old) had improved attitude of sleeping under LLINs compared to younger classes between 15–25 yrs old (P < 0.0001) but such acknowledgeable attitude was not observed among classes older than 46 yrs and above (P = 0.12776) (Table 4).

**Table 4:** Logistic regression assessing factors associated with LLIN use

**Factors**	**Number of individuals**	**Number of individual using LLIN (%)**	**Odds ratios (95% CI)**	**P**
Total number of individuals	27794	23575(84.82)		
**Place of residence**				
Rural	9458	15640(85.30)	1	
Urban	18336	7935(83.90)	0.90 [00.84-00.96]	0.00224
**Size of the household**				
0-1 member	145	131(90.34)	1	
2-4 members	3915	3490(89.14)	0.97 [00.53-01.64]	0.91232
5-7 members	8876	7692(86.66)	0.89 [00.49-01.50]	0.68577
8 and more members	14858	12262(82.53)	0.80 [00.44-01.35]	0.43840
**Age**				
15-25 years	2523	2143(84.94)	1	
26-35 years	7207	6385(88.59)	1.50 [01.31-01.72]	< 0.001
36-45 years	7741	6721(86.82)	1.34 [01.17-01.53]	< 0.001
46 and mores years	10323	8326(80.65)	0.91 [00.80-01.03]	0.12776
**Ratio 1 net for 2 person met**				
No	13174	10333(78.43)	1	
Yes	14620	13242(90.57)	2.47 [02.30-02.65]	< 0.001
**Gender**				
Female	10161	8630(84.76)	1	
Male	17633	14945(84.93)	1.10 [01.03-01.19]	0.855

In areas where the recommended ratio of 1 LLIN per every 2 persons was met, the odds of effective use increased 2.4-fold [02.30-02.65] (P < 0.001) compared to areas where this statement was not met.

We observed no influence of the gender status of the head of household in the rate at which LLINs was effectively used; the odds of usage being similar whether the household was headed by a man or woman (OR = 1.10 [01.03-01.19]; P = 0.855).

## Discussion

The study revealed that 86.4% of households surveyed have at least one LLIN and confirmed the results observed in the Demographic Health survey conducted in 2012 in Benin [12]. In another study conducted in Benin in 2010 [13] before the distribution campaign, 40% of households have at least one LLIN. These observations showed that the national distribution campaign of LLINs conducted in Benin in July 2011 has significantly increased LLINs coverage. Universal coverage at country level as defined by World Health Organization [14] is then reached. The results also confirmed the evolution of performance indicators to the strategic objectives of the sub-regions and those of the NMCP/Benin, which aimed by this free distribution campaign to bring the proportion of households with at least one LLIN from 58 to 90% by the end of 2011 [15]. Although the campaign increased household LLIN ownership, it failed to attain its goal to bring the proportion of 90% of household with at least one LLIN because the average LLIN coverage observed in this study was 86.4%. In addition, the universal bed net coverage goal defined as one for every 2 people was not also achieved. Only 77% of the households surveyed met this cut-off suggesting that the number of LLIN distributed may not be sufficient to protect and cover 23% of the Beninese households that received at least one LLIN. But, there were important variations with this indicator between departments; the proportion of households in a department that have enough nets for every two persons ranged from 52% to 86% suggesting that households that did not have enough nets ranged from 14% to 48%. In fact, the maximum number of LLINs to households in the national distribution campaign was eight per household and this may affect ownership in large households size [15]. This result was observed in north-west Tanzania after national distribution campaign [16]. The same result (40%) was also reported after national distribution campaign at Senegal in 2010 [17].

The proportion of households that received a coupon was 89% for the whole country and the proportion of household that received at least one LLIN was 86%. This observation showed that the distribution procedure was efficient but could be improved. However, the relationship between distributed coupons and receiving LLINs remains to be explored in several localities [18]. Some households, for reasons of unavailability or difficulties in finding the location of the distribution, or late removal sites distribution and also the long waiting times, have not received LLINs. This state of affairs could justify the low coverage observed in the departments of Littoral and Oueme.

In urban areas, the number of households with at least one LLIN was also lower than in rural areas. This could be associated with the fact that many people in urban areas have not time to go to the distribution point or have not received a coupon. In rural areas of Benin, people are often available and can easily go to the distribution point to receive LLIN. These reasons could explain why the place of residence plays a key role in LLIN ownership. The size of household is also associated with LLIN ownership and is adequacy with the objectives of the distribution campaign which aimed to provide LLIN according to household size. The presence of a pregnant woman did not play a role in LLIN ownership. This observation could be explained by the few number of pregnant women recorded per household in this study. The presence of children under five in the household was significantly bound with LLIN ownership. This result may be due to the presence of an average of one child under five per household. The odd of male head of households with at least one LLIN was significantly higher than that of female. This is due to the high number of male heads of household in Benin. Before the distribution campaign of 2011, to be a pregnant woman was an important factor in LLIN ownership [15]. Currently, this trend is being reversed in Benin by the distribution in universal access; but to be a child under five seems to still play a key role in LLIN ownership.

Around 84% of households’ members slept under LLIN the night before, suggesting that the distribution campaign increased bed net usage. But the number of individuals who slept under LLIN in urban areas is lower than in rural areas. This shows a low use of LLIN in urban areas and could be explained by hot weather associated with low mosquito nuisance which did not motivate LLIN use. According to Pulford *et al.*[19], low mosquito density is the most widely identified reason for LLIN non-use. However, more investigation to determine factors associated with low LLIN use in urban areas is important to induce behaviour change in urban areas. This study showed that household size does not play a role in LLIN use. LLIN use was similar among family members [20] showing that the 2011 distribution campaign successfully achieved the goal of universal access to LLIN. All groups of people at risk to malaria infection are covered but the middle age class (25–46 yrs old) had an improved attitude of sleeping under LLINs. This finding was also observed by Garley *et al.*[21] in Nigeria who showed that people over 25 years old use more LLIN. This difference in LLIN use between this age group and others could be due to sleeping arrangements. People of this class of age are often married and sleep together with their spouse; this could increase the number of people of this class of age that use LLIN comparatively to other class of age.

The universal coverage aimed to cover all people in the community but not only the target groups. The goal is to provide equitable protection to all household members and to benefit of the collective protecting effect induced by the high household coverage. The comparison of LLIN usage between family members illustrated the increased conscientiousness in LLIN use in Beninese population. Then, with more efforts and engagements from different partners implicated in malaria control strategies in Benin, all the goals of universal coverage will be achieved.

This study showed positive impact of mosquito net distribution campaign in Benin. However, further assessment would have been possible, if logistics and baseline data for households’ economic characteristic and net use were available. The analysis of net coverage and net use was restricted to the post campaign survey and reference before the campaign was also limited to PNLP (2011) [13]. Therefore, it was important to conduct an assessment before each distribution campaign to provide reference data for the post-campaign assessment.

## Conclusion

This study shows an improvement of the performance indicators towards universal coverage. But it should be clarified that further efforts are still required to achieve the standard of an ITN for two people in the household. Efforts are also required in order to maintain the observed coverage rates in different department safer this campaign that help to cover around nine out of ten households.

This assessment allows us to have the data that could help the national malaria control program to improve others LLIN campaigns to reduce morbidity and mortality due to malaria in Benin.

## Competing interests

There are neither any financial competing interests nor any non-financial competing interests (political, personal, religious, ideological, academic, intellectual, commercial or any other) to declare in relation to this manuscript.

## Authors’ contributions

FTT collected analyzed, interpreted data and wrote the manuscript. BA EYD and AB were responsible for field collection; Data analyze and helped in drafting the manuscript. VG contributed to the mapping, helped in drafting the manuscript and revised the manuscript. RN interpreted data and revised the manuscript. MO, DKF and MCA conceived and designed the study, supervised field’s procedures, and review the manuscripts. All authors have read and approved the manuscript.
